# Outcomes of Providing Children Aged 7-12 Years With Access to Evidence-Based Anxiety Treatment Via a Standalone Digital Intervention Using Immersive Gaming Technology: Real-World Evaluation

**DOI:** 10.2196/52866

**Published:** 2024-10-22

**Authors:** Brioney Gee, Bonnie Teague, Andrew Laphan, Tim Clarke, Georgianna Coote, Jessica Garner, Jon Wilson

**Affiliations:** 1 University of East Anglia, Norwich, United Kingdom; 2 Norfolk and Suffolk NHS Foundation Trust, Norwich, United Kingdom; 3 Eastern Academic Health Sciences Network, Cambridge, United Kingdom

**Keywords:** anxiety, children, young people, exposure therapy, graded exposures, cognitive behavioural therapy, digital intervention, mobile app, gaming, real-world evaluation, gaming technology, real-world implementation

## Abstract

**Background:**

Anxiety disorders are among the most common mental health conditions in childhood, but most children with anxiety disorders do not access evidence-based interventions. The delivery of therapeutic interventions via digital technologies has been proposed to significantly increase timely access to evidence-based treatment. Lumi Nova (BfB Labs Limited) is a digital therapeutic intervention designed to deliver evidence-based anxiety treatment for those aged 7‐12 years through a mobile app incorporating immersive gaming technology.

**Objective:**

We aimed to evaluate the real-world impact of providing access to Lumi Nova through UK National Health Service–funded mental health services.

**Methods:**

We analyzed precollected anonymized data routinely captured through the implementation of Lumi Nova from children aged 7‐12 years, who lived in the United Kingdom and had the opportunity to use the intervention for at least 1 week over an 18-month period. Engagement indices included whether the game key was activated, number of unique sessions, time spent engaging, and number of “challenges” completed. Clinical outcomes were assessed using the Goal-Based Outcomes measure and Child Outcome Rating Scale. Demographic data were analyzed to assess the health equality implications of Lumi Nova.

**Results:**

Of 1029 eligible families invited to use Lumi Nova, 644 (62.5%) activated their game key, of whom 374 (58.1%) completed at least one in-game graded exposure challenge. The median number of unique sessions was 6 (IQR 3‐12) and the median time spent engaging with the intervention was 42 (IQR 15‐79) minutes. For the subset of young people with paired outcomes, there were statistically significant small to medium improvements in goal-based outcome scores (n=224; *t*_223_=5.78, *P*<.001; *d*=0.37, 95% CI 0.25‐0.52) and Child Outcome Rating Scale scores (n=123; *t*_122_=5.10, *P*<.001; *d*=0.46, 95% CI 0.27‐0.65) between the first and last data points. Two in 5 young people’s scores reflected a change that would be considered reliable. Analysis of demographic characteristics tentatively suggested that children from ethnic minority backgrounds and those living in the most deprived neighbourhoods may be less likely to access Lumi Nova, but children from socioeconomically deprived areas were more likely to successfully complete a challenge once they accessed the intervention (*P*=.02). However, the level of missing data and small number of children in some demographic groups limited meaningful statistical comparisons.

**Conclusions:**

This study provides initial evidence that Lumi Nova may be associated with improved outcomes for those aged 7‐12 years seeking anxiety treatment in real-world settings. However, the lack of a control comparator group and information about concurrent treatments accessed by the young people, in addition to substantial attrition, limited the analysis that could be conducted and confidence in the conclusions drawn.

## Introduction

### Access to Evidence-Based Treatment for Children Experiencing Mental Health Problems

Evidence suggests that levels of mental ill-health among children and young people are rising, exacerbated in recent years by the COVID-19 pandemic [1]. In England, 16.7% of children aged 5-6 years were identified as having a probable mental health disorder in 2022, increasing from 12.1% in 2017 [2]. Providing timely access to evidence-based treatment to the growing number of children and young people seeking care from mental health services poses an urgent public policy challenge [3]. Given significant shortages in the mental health workforce [4], innovative approaches are required to meet this challenge.

The delivery of therapeutic interventions via digital technologies has been proposed as 1 innovation with the potential to significantly increase access to evidence-based treatment for young people [5]. Systematic reviews examining the effectiveness of internet-based or digital cognitive behavioral therapy (CBT) support the view that these digital health interventions are effective and feasible to treat depression and anxiety in children and young people, although the quality and depth of the research field has been questioned [6-8]. Meta-analytic evidence suggests that digital CBT programmes can produce similar outcomes to face-to-face therapy [9,10]. Further, given that many children are comfortable and familiar with using digital devices (eg smartphones and tablets), young people may find this mode of delivery particularly appealing [11].

However, this research evidence has not established if digital therapies based on CBT work for all young people equitably. For example, children from disadvantaged and marginalized groups are more likely to experience poor mental health than their peers [12,13], while also being disproportionately affected by obstacles to accessing health care [14]. There is potential for digital interventions to promote equity in mental health outcomes by reducing disparities in access to care between young people of different backgrounds [15]. However, it is also important to remain cognisant of the potential for new technologies to exacerbate existing health inequalities [16] and to ensure that any such impacts can be mitigated against.

Of particular focus to this paper, there is limited evidence for the use of digital health interventions for young people’s anxiety. Anxiety disorders are among the most common mental health conditions in childhood. Prevalence rates from 2003‐2020 were estimated to be around 5% in high-income countries before the COVID-19 pandemic [17], and appear to have increased following the pandemic [18]. Childhood anxiety disorders can have significant negative impact on educational attainment, peer relationships, and family life, and often persist into adulthood if left untreated [19]. However, at present, most children with anxiety disorders do not access evidence-based interventions. In a UK study, of the 65% of families of children with anxiety who sought support, only 38% accessed support and less than 3% accessed an evidence-based treatment [20]. While many mobile-based applications designed to help children with anxiety have been made available on consumer marketplaces, very few are underpinned by core therapeutic principles for the treatment of anxiety disorders or have been subject to empirical evaluation [21]. This paper will be examining one of these digital applications, Lumi Nova, designed to support young people’s anxiety in real-life clinical settings.

### Lumi Nova: Tales of Courage

Lumi Nova: Tales of Courage (“Lumi Nova”) is a standalone digital therapeutic intervention designed to deliver evidence-based anxiety treatment for those aged 7‐12 years [22]. It provides graded exposure therapy (a key active ingredient of CBT for anxiety [23]) and psychoeducation via an immersive, engaging app-based mobile game. Lumi Nova was launched by BfB Labs in September 2020 following a 3-year development process led by a human centred design approach. This process involved engagement with academics, clinicians, service managers, commissioners, games experts, education professionals, parents, and children. The aim of these development phases was to ensure the intervention has both a robust clinical basis and high-end functionality, incorporating the latest advances in immersive gaming technology to increase voluntary engagement. A preliminary evaluation found that Lumi Nova is safe and has the potential to benefit children experiencing mild to moderate anxiety [24]. Current guidance for the use of Lumi Nova in the home recommends that young people use the intervention for up to 30‐40 minutes per day and are asked to select 3 goals that they would prefer to work on. These goals include working on personalized challenges selected by the young person, such as “be comfortable speaking in a group,” “be able to sleep away from home overnight,” or “feel comfortable going to school” [25]

The young person uses the intervention on their own, although they are supported by their parent or guardian to access the intervention on a device, who is notified when the young person accesses the intervention. In practice, the number of sessions and frequency of use is determined by the young person. Demographic, usage, and outcome data from 2 measures is collected on an embedded secure digital platform.

Young people presenting to the UK National Health Services (NHS) with anxiety are offered the opportunity to use Lumi Nova after initial screening in person or via telephone, or during later treatment appointments. Some families are supported to access the intervention by a practitioner, while others are given access via a promoted “self-sign-up” option (implemented from April 2022 onwards) which allows the child’s parent or caregiver to access the intervention independently.

Young people are given access to the game either as a stand-alone treatment for mild to moderate anxiety, alongside other therapeutic support, or while on a waitlist for further support.

Lumi Nova is currently in use by providers of children and young people’s mental health services across multiple regions of the United Kingdom, including both NHS and voluntary sector providers. The software is CE (Conformité Européene) marked and registered with the UK’s Medicines and Healthcare Products Regulatory Agency as a medical device (class 1—low risk). Lumi Nova has recently been recommended by the National Institute for Health and Care Excellence in their Early Value Assessment of digital CBT for children and young people with mild to moderate symptoms of anxiety or low mood [26]. This assessment recommended that Lumi Nova be used as a first line treatment option for children and young people with mild to moderate anxiety difficulties but suggested that this usage should be accompanied by further evidence generation efforts.

### Objectives

The current evaluation begins to address this National Institute for Health and Care Excellence recommendation to generate further evidence by using routinely captured engagement and outcome data to better understand how Lumi Nova has been used in real-world settings. Using this data, we were able to investigate (1) the rate and degree of engagement with Lumi Nova by young people who were provided with access to the intervention as part of their routine care over an 18-month period, (2) the clinical outcomes for young people provided with access to Lumi Nova over this period, and (3) whether engagement or outcomes for young people from disadvantaged or marginalized groups differ from those of their peers.

## Methods

### Design

We conducted a real-world evaluation of Lumi Nova using precollected quantitative data routinely captured by BfB Labs. User engagement and outcome data are collected by BfB Labs for the purposes of enabling families and professionals responsible for the care of young people using Lumi Nova to monitor the child’s progress via the product’s dashboard, as well as to enable evaluation and iterative improvement of their products and services. Relevant data were exported from the product’s dashboard and anonymized by a member of BfB Labs team before being securely transferred to the research team for analysis.

This evaluation was part of a wider study investigating the utility of digital therapeutic interventions within NHS-funded children and young people’s mental health services (the Investigating Digital Therapy study). This study aimed to provide insights into the potential for digital technologies to improve access to evidence-based treatment, to explore possible impacts on health inequalities, and identify factors that impact the commissioning and implementation of new digital technologies within the sector.

### Participants

Young people whose precollected data were eligible for inclusion in the analysis were those who (1) had been referred by practitioners to use Lumi Nova during an 18-month period from July 2021 (when the current outcome measures were implemented) to December 2022, (2) had the opportunity to use the intervention for at least one week, (3) were aged 7‐12 years, and (4) who were residing in the United Kingdom. No additional or new data was collected for analysis.

### Measures

Demographic information collected from Lumi Nova users included the child’s age, gender, ethnicity, disability status, and home postcode. Postcodes were used to classify the relative deprivation of individuals living in that area using the indices of multiple deprivation, which categorizes small areas of England, Wales, and Scotland from most (1) to least (10) deprived based on a combination of 37 indicators [27].

User data collected to provide a measure of engagement included: whether each family invited to access the game went on to activate their game key, the number of unique sessions completed, the amount of time spent engaging with the intervention (total length of sessions minus any idle time), and the number of “challenges” completed as part of the game (including both in-application challenges and real-world challenges completed outside of the application and verified as complete by the child’s parent or caregiver).

Clinical outcome data was collected using the goal-based outcomes (GBOs [28]) measure and the Child Outcome Rating Scale (CORS [29]). GBOs are a method of quantifying progress toward the goals a young person sets for themselves at the beginning of a clinical intervention. Scoring range is 0‐10, with higher scores indicating greater perceived progress toward the selected goals. Children were asked to complete this measure within the application after selecting their goals for the first time and then on a weekly basis (if the child logs into the application) before completing a challenge.

The CORS is a 4-item parent-reported measure developed to enable monitoring of a child’s symptom distress, interpersonal well-being, social role functioning, and overall well-being on a regular basis during the process of therapeutic interventions. Scoring range is 0‐40, with higher scores indicating better functioning. The child’s parent or caregiver was first asked to complete the CORS during the process of accessing the application, before being provided with a game key. The child’s parent or caregiver was subsequently prompted to repeat the measure weekly via a link sent to their mobile using SMS messaging; if the measure was not completed, 2 reminders were sent via SMS.

Young people were also asked to rate how anxious they felt before the challenge and, after completing the challenge, how anxious they felt during the challenge, and how anxious they would feel if they had to complete the challenge again on a 1 to 5 scale 5 being most anxious) via in-game ratings. Ratings of anxiety before and during the challenge were used as an indication of mismatch in anticipated and experienced anxiety, which has been posited as an important mechanism through which graded exposure therapy brings about reductions in anxiety symptoms [23].

### Data Analysis

Descriptive statistics were calculated for all demographic characteristics, engagement indices, and outcome measures. After confirming all relevant assumptions were met, paired sample *t* tests were conducted to assess whether changes in outcome measures were statistically significant at the *P*=.05 level (GBOs were treated as the primary outcome and corrections for multiple comparisons used for other measures). Standardized effect sizes (Cohen *d*) and their 95% CIs were calculated to quantify the size of statistically significant differences. Only cases for which completed outcome measures were available at 2 or more time points were included in outcome assessments. Due to high levels of attrition and considerable variation in the time points of available follow-up data, comparisons were made between the first and last available data point for each participant, regardless of the spacing of these data points. Further, we focused on the first goal selected by the user to work toward only. Bivariate correlation coefficients were calculated to explore any associations between engagement indices and outcome change scores.

To assess any differences in access between demographic groups, the numbers of children invited to use the intervention and who activated their game key were disaggregated by gender, ethnicity, disability status, and neighbourhood deprivation. This information was compared with available data from the 2021 census in England and Wales to assess how closely the cohort of young people accessing Lumi Nova reflects the wider population of those aged 7‐12 years.

To explore any difference in engagement or outcomes between groups, *χ*^2^ tests were used to assess whether there are statistically significant between-group differences in activation or number of challenges completed, and mixed-effect ANOVAs were used to assess whether there is statistically significance between-group differences in mean change over time in GBO or CORS scores.

### Patient and Public Involvement

Patient and public involvement has been central to BfB Labs’ approach to designing, building, and testing digital interventions for young people. From the outset BfB Labs have worked alongside their target users to codesign interventions, using nonextractive iterative, user-led, agile design methodology. During this study, we worked alongside a panel of young advisors who advised on all aspects of this study, contributed to the interpretation of study findings, and supported dissemination efforts. Young advisors were paid for their time and offered opportunities to take part in training to build their research skills, as well as letters of recommendation or references to support their educational or professional development. The opportunity to be named as coauthors on this paper were discussed with the group, but by mutual agreement, young advisors wished to be acknowledged in this paper only.

### Ethical Considerations

Ethical approval was obtained from the NHS Health Research Authority following a favourable ethical opinion from North West—Preston Research Ethics Committee (22/NW/0195). As the data were routinely collected and analysed retrospectively, study specific consent was not sought for reasons of practicality and limiting burden on users. However, parents or caregivers were informed that anonymized user data from the application would be used to evaluate the intervention and indicated their agreement to their child’s data being used for this purpose during the sign-up process. Only approved staff members from BfB Labs had access to patient identifiable data, which was anonymized before analysis by the research team; the research team were not granted access to the anonymized data until confirmation of Health Research Authority approval for the evaluation was received.

## Results

### Participants

The initial dataset included data from 1202 families invited to use Lumi Nova during the period of interest. During the data cleaning process, we excluded 173 families from the evaluation who did not appear to meet the eligibility criteria (based on their responses to demographic questions or date of invitation or activation). Reasons for exclusion were: not having had the opportunity to use the intervention for at least one week (n=22), being outside of the target age range for the game (n=147), and not currently living in the United Kingdom (n=4). Demographic characteristics of the sample are summarized in Table 1.

**Table 1.** Sample characteristics.

Characteristics		All cases (n=1029)	Activated cases (n=644)
Age (years), mean (SD, range)		9.71 (1.46, 7.00‐12.98)	9.72 (1.44, 7.00‐12.98)
**Gender**			
	Female, n (%)	492 (51.79)	312 (52)
	Male, n (%)	458 (48.21)	288 (48)
	Missing or not available, n	79	44
**Ethnicity**			
	Arab, n (%)	1 (0.1)	1 (0.2)
	Asian or Asian British, n (%)	12 (1.4)	9 (1.7)
	Black, African, Caribbean, or Black British, n (%)	17 (2)	7 (1.3)
	Chinese, n (%)	1 (0.1)	1 (0.2)
	Mixed or multiple ethnic group, n (%)	45 (5.3)	23 (4.4)
	Other ethnic group, n (%)	8 (0.9)	4 (0.8)
	White, n (%)	772 (90.19)	480 (91.42)
	Missing, n	173	119
**Disability**			
	True, n (%)	122 (11.86)	76 (11.8)
	False, n (%)	907 (88.14)	568 (88.2)
**Referral type**			
	Self–sign up, n (%)	618 (60.05)	381 (59.16)
	Clinician referral, n (%)	411 (39.95)	263 (40.84)
**Operating system**			
	Android, n (%)	—^a^	219 (34)
	iOS, n (%)	—	425 (66)

a N/A: not applicable.

### Engagement

**Figure 1.**
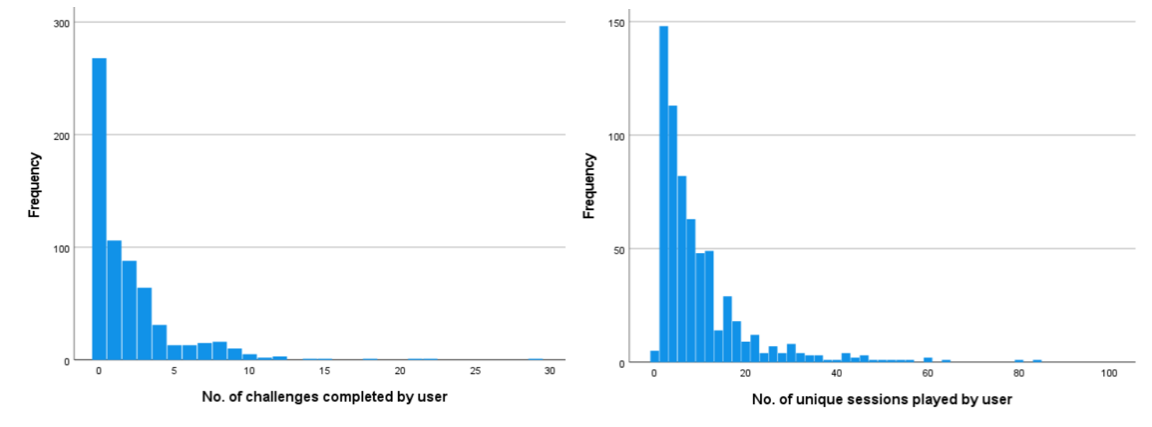
Histograms showing distribution of engagement indices.

Of the 1029 families included in the final dataset, 644 (62.58%) parents or caregivers completed the baseline CORS to activate their Lumi Nova game key to enable their child to use the intervention. As the distributions of all engagement indices were positively skewed, medians and IQRs were calculated. The median number of unique sessions completed by those who activated their game key was 6 (IQR 3‐12). The median total time spent engaging with the intervention was 42 (IQR 15‐79) minutes and the median number of challenges completed was 1 (IQR 0‐3); 58.1% (n=358) completed at least one challenge, 42% at least two (n=270), 28.2% at least three (n=181), and 17.7% (n=114) completed 4 or more. Those who completed at least one challenge also engaged in the psychoeducation element of the intervention as this is a prerequisite of commencing the first challenge. Histograms showing the distribution of unique sessions and challenges completed are in Figure 1.

### Outcomes

Paired GBO data was available for 224 cases and paired CORS data for 123 cases. There was a statistically significant increase in GBO scores between the first and last available data point (*t*_223_=5.78, *P*<.001). The size of the effect was in the small to medium range (*d*=0.37, 95% CI 0.25‐0.52), indicating that, on average, young people who engaged with the game made progress toward their selected goal. At the individual level, 39.3% (n=88) of cases saw a reliable improvement in GBO scores, 14.3% (n=32) a reliable deterioration, and 46.4% (n=104) had no reliable change.

There was also a statistically significant increase in CORS scores between the first and last available data point (*t*_122_=5.10, *P*<.001). The size of the effect was in the small to medium range (*d*=0.46, 95% CI 0.27‐0.65), indicating that parents or caregivers of children who engaged with the game reported improvements in functioning. At the individual level, 37.34% (n=46) of cases saw a reliable improvement in CORS score, 9.8% (n=12) a reliable deterioration, and 52.8% (n=65) had no reliable change.

Paired anxiety ratings pre and post at least one challenge were available for 363 cases. The average anxiety rating prechallenge was statistically significantly higher than the average postchallenge rating (*t*_361_=8.35, *P*<.001). The size of the effect was again in the small to medium range (*d*=0.44, 95% CI 0.33‐0.55). There was no association between the size of the average discrepancy between pre and post challenge anxiety ratings and change in GBO or CORS scores.

No associations were evident between the number of challenges completed or time spent engaging with the intervention and change score for either the GBO, CORS, or average anxiety ratings pre and post challenges.

### Equality of Access, Engagement, and Outcomes

Of those young people whose parent or caregiver activated their game key, 51.79% (492/1029) identified as female and 48.21% (458/1029) as male. Females were significantly more likely to complete at least one challenge than males (n=200, 61.9% vs n=158, 53.5%, *P*=.02). There were no other significant differences between males and females in markers of engagement or outcomes.

More young people whose parent or caregiver activated their game key identified as White (480/644, 91.42%) than the percentage of those aged 7‐12 years who identify as White in the general population of England and Wales (n =3,174,513, 73.5%). Conversely, children of Asian and Black ethnicities were underrepresented among Lumi Nova users compared to the population of those aged 7‐12 years in England and Wales (n=12, Asian 1.7% vs n=486,124, 11.2% and Black n=17, 1.3% vs n=237,872, 5.5% respectively). It was not possible to conduct meaningful statistical analyses of any differences in engagement or outcomes between ethnic groups due to the small number of Lumi Nova users in the sample who identified as belonging to each group. Further, it should be noted that, due to commissioning arrangements, Lumi Nova was only available in certain areas which may not have reflected the demographics of the country as a whole, and often only to those already under the care of mental health services.

Compared to the general population of those aged 7‐12 years, Lumi Nova users were more likely to live in the least deprived areas of the United Kingdom and less likely to live in the most deprived areas. However, young people living in the most deprived areas were significantly more likely than those living in more affluent areas to complete at least one challenge (n=87, 66.4% vs n=125, 53.8%; OR 1.68, 95% CI 1.08-2.64, *P*=.02). There were no significant differences in the outcomes of young people living in the most deprived areas compared to the most affluent areas.

Of those young people whose parent or caregiver activated their game key, 11.8% (n=121) reported a disability, a higher percentage with a disability than in the general population of those aged 7‐12 years (n=190,287-445,469, 4.4%‐10.3%). However, young people with disabilities were less likely to successfully complete at least one challenge than nondisabled users (n=32, 42.7% vs n=326, 59.6%, OR 0.5, 95% CI 0.3-0.81, *P*=.005). The number of young people with a disability for whom paired outcome data was available was small (GBOs, n=20; CORS, n=11). However, within this small cohort of users, improvements in GBO and CORS scores were smaller than for nondisabled young people. The nature of reported disabilities (ie, physical, learning, or neurodevelopmental) was not recorded.

## Discussion

### Principal Findings

There is a pressing need to improve young people’s access to evidence-based mental health treatments. To meet this need in the context of growing demand for mental health support and a limited pool of qualified practitioners, innovative approaches are required, including improved use of digital technologies. Lumi Nova, a digital therapeutic intervention which uses immersive gaming technology to deliver an evidence-informed anxiety intervention for those aged 7‐12 years, is an example of an innovation with the potential to facilitate increased access to timely treatment. We conducted a real-world evaluation of Lumi Nova, to better understand user engagement and outcomes, and to explore whether these might differ across demographic groups.

We found that the majority (n=644, 62.58%) of families invited to access Lumi Nova activated their game key, of whom 58.1% (n=358) completed at least one challenge. However, only a relatively small proportion of users went on to complete multiple challenges, resulting in a median number of challenges completed of 1 (IQR 0‐3). The median number of unique sessions was 6 (IQR 3‐12), but these sessions were typically short relative to the usual duration of face-to-face therapy sessions, with the median total time spent engaging with the intervention being 42 (IQR 15‐79) minutes.

For the subset of young people for whom paired outcome measures were available, there were statistically significant small to medium improvements in perceived progress toward goals and parent-reported functioning between the first and last data points. Two in 5 young people reported an improvement that would be considered reliable (ie, the magnitude of the change is such that it is unlikely to be accounted for by measurement error) for both the GBOs and CORS. Available paired anxiety ratings suggest that young people’s anticipated anxiety prechallenge was, on average, higher than the anxiety they reported post challenge, providing some support that the mechanisms of graded exposure were operating as would be anticipated. However, there was no evidence of an association between either the extent of mismatch in anxiety expectation or the child’s level of engagement and the outcomes observed.

Interpretation of these findings is complicated by the small proportion of the overall sample for whom paired data was available, the uncontrolled design and limited available comparison data from studies of interventions delivered in real-world treatment settings. However, analysis of routinely collected data from the Child Outcomes Research Consortium [30] suggests that the outcomes observed for young people accessing Lumi Nova are broadly in line with those observed in young people receiving routine treatment for child and adolescent mental health services.

Given the relatively brief duration of the intervention received, that we observed outcomes of a comparable magnitude to those seen in routine practice could be interpreted as preliminary evidence that Lumi Nova may offer a particularly efficient treatment option. However, we are unable to rule out the possibility that the improvements observed may simply reflect natural recovery or the effect of other support received alongside Lumi Nova. Further, as paired outcome data was only available for a subset of those who activated the intervention, we cannot be sure that these findings would generalize to all Lumi Nova users, let alone the wider population of children seeking treatment for anxiety.

Comparing the demographic characteristics of Lumi Nova users to the general population of those aged 7‐12 years in England and Wales suggested that children from ethnic minority backgrounds and those living in the most deprived neighbourhoods may be less likely to access Lumi Nova than other young people. However, once users from more deprived areas did access Lumi Nova, they were more likely than affluent users to complete at least one challenge. Given that ethnic minority and economically disadvantaged young people are more likely to experience mental health difficulties than their peers [12,13], this is an important indicative finding that requires further investigation.

However, it should be borne in mind that, due to commissioning arrangements, Lumi Nova was only available in certain mental health services around the United Kingdom during the implementation period studied. These services were located in diverse areas of the United Kingdom including the East of England, London, Wales, Essex, Yorkshire, Bristol, Gloucestershire, and Kent, but only within certain organisations and specific NHS mental health teams. As such, the demographic comparative work relating to deprivation and ethnicity undertaken in this paper has used UK-wide data to reflect this diverse geography, in lieu of the absence of population data in these specific areas for those aged 7‐12 years. Furthermore, since the option for families to access the intervention via self-sign-up was implemented toward the end of the period studied, and only in certain regions, in most cases, only those already in contact with mental health services would have been offered Lumi Nova, yet no quality mental health service access data on a specific age basis is publicly available which met the needs of this study analysis. It is possible that further mitigations are needed to overcome barriers to help-seeking among marginalized groups. For instance, expansion and further promotion of the self-sign-up option may help to overcome structural and attitudinal barriers to accessing traditional mental health services [11].

When examining differences in outcomes based on user characteristics, we found some evidence that females and those from less affluent areas may be more likely than other users to complete at least one challenge, but, overall, we identified few differences between the engagement and outcomes of Lumi Nova users across demographic groups. However, the small numbers of young people in the sample who identified as belonging to some minority groups and the high volume of missing data limited the statistical comparisons that could meaningfully be made. There were preliminary indications that young people with a disability may be less likely to successfully engage with the intervention than children without a disability. The small size of those subgroups involved, alongside the limited available information about the nature of disabilities reported, precludes any firm conclusions from being drawn from this finding. However, further research investigating the accessibility of digital interventions such as Lumi Nova to young people with disabilities is warranted.

### Strengths and Limitations

One of the key strengths of this study was that it involved a real-world evaluation, reflecting pragmatic usage of the intervention as part of routine practice in NHS settings and families outside of experimental research contexts, conferring external validity to the findings [31]. This approach also allowed for analysis on a larger dataset of 1029 people, even given the extent of attrition, than would have been possible to collect in a controlled study carried out with similar resources and over a similar timeframe. Moreover, the scope of data collected and analysed in this study was more extensive than is typically made available for commercial digital interventions and services, incorporating demographic, engagement, or access and outcome data, not just data regarding initial access. Collectively, this allowed for an in depth exploration of not only whether Lumi Nova appears to be effective to treat young people’s anxiety, but an insight into the platform’s users, acceptance and real-life usage in the home, and early indicators about who it is more or less likely to benefit. These insights are important for practitioners and commissioners to consider if wishing to implement Lumi Nova into future services.

However, there were also several limitations to the approach taken in our study. Relying on retrospective routinely captured data completed by young people themselves without support by professionals meant the dataset had more missing data than would be anticipated in a prospective study design where missing data could be identified and addressed during the research process, posing some challenges during the data cleaning process. We were also limited by data relating to the population census statistics as a comparator, as no meaningful mental health service data is available for this specific age range. The UK-wide implementation of Lumi Nova, albeit across selected services only, meant that we were limited to comparisons of the user cohort with UK-level population data rather than using mental health service data or local small area census data. We were also limited by the choice of measures and other information available within the dataset. For instance, no standardized self-report measure of anxiety symptom severity was available and data on disabilities was captured as a binary variable, with no information available on the nature of the disability, which may have been relevant to understanding accessibility. Importantly, the real-life approach also meant that this study had no control comparator group and there was limited information on what (if any) treatment young people received in addition to Lumi Nova from the NHS or other care providers, compounding the limitations imposed by the uncontrolled study design when evaluating the cohort’s clinical outcomes. This means that conclusions of effectiveness need to be treated with caution as improvements relating to natural recovery or the impact of other treatment interventions delivered in the same period as Lumi Nova usage cannot be ruled out.

Further, interpretation of the findings was made more challenging by the high level of missing data. This challenge was heightened by the impossibility of distinguishing between missing data due to a child disengaging from the intervention and missing data due to a child playing the game offline following the initial download and their data not being transferred to the central data hub as a result.

### Implications and Recommendations

Providers of digital therapeutics should be encouraged to collect, monitor, and make available for analysis, data on how interventions are being used, by whom, and the outcomes observed. It should be noted that the quality and completeness of routine data collection by traditional child and adolescent mental health services is often poor [25]. As such, we must be cautious of holding digital interventions to standards of monitoring and evaluation that far exceed those of nondigital interventions. However, we should work toward thorough and systematic collection and analysis of routine data across all forms of child and adolescent mental health treatment.

Learning from this evaluation has already been used by BfB Labs to refine their processes to improve the quality and completeness of the data they are able to capture. For instance, extra data save points have been implemented to minimize the loss of data in the event the child does not continue to the next save point or connect to the internet for their data to be uploaded. This improved data will be used by BfB Labs to continue to evaluate and refine their product.

Further research is needed to explore how any barriers to access or engagement can be reduced among those with disabilities, young people from marginalized ethnic backgrounds, and those living in the most deprived areas to ensure all young people can benefit equally from interventions such as Lumi Nova. We are aware of an ongoing study (ISRCTN Registry, ISRCTN11131689) specifically aiming to understand the barriers to uptake and usage of Lumi Nova among children growing up in circumstances of economic disadvantage. This study aims to understand how the intervention can be implemented to maximize usage and engagement among those living in postcodes in the 2 most deprived deciles of England. Future research should also evaluate the benefits and costs of offering Lumi Nova and other digital therapeutic interventions via self-sign-up routes versus requiring families to be on-boarded by mental health clinicians.

On a broader level, this study also raises methodological questions regarding the best way to provide evidence for digital therapeutic interventions. Digital interventions must continually and rapidly evolve if they are to keep pace with technological advancements, compatibility issues, and user expectations. As such, there is a need to ensure that approaches to evaluation balance scientific rigour with the ability to provide evidence that is timely and has sufficient ecological validity to retain relevance to real-world settings. Researchers, commissioners, providers, guidance authors, and innovators must work together to agree on what constitutes a sufficiently robust approach to evaluation in this rapidly developing field.

### Conclusions

Providing the growing number of children seeking mental health support with timely access to care poses a significant challenge. This study provides indicative evidence that use of Lumi Nova may be associated with improvements in functioning and goal-based outcomes among those aged 7‐12 years seeking treatment for anxiety difficulties. However, the strength of this study’s findings is limited by a lack of a control comparator group and missing information about any concurrent treatments being received by young people using the intervention. As such, Lumi Nova has the potential to play an important role in improving access to mental health treatment for children experiencing anxiety. However, further research is needed to confirm these findings as natural recovery or the impact of other interventions cannot be ruled out. Future research should consider how engagement with the intervention can be maximized and sustained where beneficial for that child’s needs, particularly in children from less advantaged groups.

## Abbreviations

CBT: cognitive behavioral therapy
CE: Conformité Européene
CORS: Child Outcome Rating Scale
GBO: goal-based outcome
NHS: National Health Service

## References

[1] Deng J, Zhou F, Hou W (2023). Prevalence of mental health symptoms in children and adolescents during the COVID-19 pandemic: a meta-analysis. Ann N Y Acad Sci.

[2] (2022). Mental health of children and young people in England 2022 - wave 3 follow up to the 2017 survey. NHS Digital.

[3] Neufeld SAS (2022). The burden of young people’s mental health conditions in Europe: no cause for complacency. Lancet Reg Health Eur.

[4] British Medical Association (2020). Measuring progress: commitments to support and expand the mental health workforce in england. https://www.bma.org.uk/media/2405/bma-measuring-progress-of-commitments-for-mental-health-workforce-jan-2020.pdf.

[5] Schueller SM, Torous J (2020). Scaling evidence-based treatments through digital mental health. Am Psychol.

[6] Eilert N, Enrique A, Wogan R, Mooney O, Timulak L, Richards D (2021). The effectiveness of internet-delivered treatment for generalized anxiety disorder: an updated systematic review and meta-analysis. Depress Anxiety.

[7] Hollis C, Falconer CJ, Martin JL (2017). Annual research review: digital health interventions for children and young people with mental health problems - a systematic and meta-review. J Child Psychol Psychiatry.

[8] Lehtimaki S, Martic J, Wahl B, Foster KT, Schwalbe N (2021). Evidence on digital mental health interventions for adolescents and young people: systematic overview. JMIR Ment Health.

[9] Ebert DD, Zarski AC, Christensen H (2015). Internet and computer-based cognitive behavioral therapy for anxiety and depression in youth: a meta-analysis of randomized controlled outcome trials. PLoS One.

[10] Pennant ME, Loucas CE, Whittington C (2015). Computerised therapies for anxiety and depression in children and young people: a systematic review and meta-analysis. Behav Res Ther.

[11] Silk JS, Pramana G, Sequeira SL (2020). Using a smartphone app and clinician portal to enhance brief cognitive behavioral therapy for childhood anxiety disorders. Behav Ther.

[12] Abraham A, Walker-Harding L (2022). The key social determinants of mental health: their effects among children globally and strategies to address them: a narrative review. Pediatr Med.

[13] Reiss F, Meyrose AK, Otto C, Lampert T, Klasen F, Ravens-Sieberer U (2019). Socioeconomic status, stressful life situations and mental health problems in children and adolescents: results of the German BELLA cohort-study. PLoS One.

[14] Robards F, Kang M, Usherwood T, Sanci L (2018). How marginalized young people access, engage with, and navigate health-care systems in the digital age: systematic review. J Adolesc Health.

[15] Piers R, Williams JM, Sharpe H (2023). Review: can digital mental health interventions bridge the ‘digital divide’ for socioeconomically and digitally marginalised youth? A systematic review. Child Adolesc Ment Health.

[16] Middle R, Welch L (2022). Experiences of digital exclusion and the impact on health in people living with severe mental illness. Front Digit Health.

[17] Barican JL, Yung D, Schwartz C, Zheng Y, Georgiades K, Waddell C (2022). Prevalence of childhood mental disorders in high-income countries: a systematic review and meta-analysis to inform policymaking. Evid Based Mental Health.

[18] Creswell C, Shum A, Pearcey S, Skripkauskaite S, Patalay P, Waite P (2021). Young people’s mental health during the COVID-19 pandemic. Lancet Child Adolesc Health.

[19] Hill C, Waite P, Creswell C (2016). Anxiety disorders in children and adolescents. Paediatr Child Health (Oxford).

[20] Reardon T, Harvey K, Creswell C (2020). Seeking and accessing professional support for child anxiety in a community sample. Eur Child Adolesc Psychiatry.

[21] Bry LJ, Chou T, Miguel E, Comer JS (2018). Consumer smartphone apps marketed for child and adolescent anxiety: a systematic review and content analysis. Behav Ther.

[22] BfB Labs Lumi Nova: tales of courage. https://www.bfb-labs.com/luminova.

[23] Furnham A, Wilson E, Chapman A, Persuad R (2013). Treatment hurts: lay theories of graded exposure in the treatment of four anxiety disorders. Eur J Psychother Couns.

[24] Lockwood J, Williams L, Martin JL, Rathee M, Hill C (2022). User engagement and experience, and safety of a mobile app (Lumi Nova) delivering exposure-based cognitive behavioral therapy strategies to manage anxiety in children via Immersive gaming technology: preliminary evaluation study. JMIR Ment Health.

[25] (2023). Guided self-help digital cognitive behavioural therapy for children and young people with mild to moderate symptoms of anxiety or low mood: early value assessment. National Institute for Health and Care Excellence.

[26] (2021). List of goals and challenges. Lumi Nova: Tales of Courage.

[27] (2019). English indices of deprivation 2019. UK.gov/Ministry of Housing, Communities & Local Government.

[28] Law D, Jacob J (2015). Goals and Goal Based Outcomes (GBOs).

[29] Campbell A, Hemsley S (2009). Outcome rating scale and session rating scale in psychological practice: clinical utility of ultra-brief measures. Clin Psychol.

[30] Wolpert M, Jacob J, Napoleone E, Whale A, Calderon A, Edbrooke-Childs J (2016). Child-and parent-reported outcomes and experience from child and young people’s mental health services 2011–2015. Child Outcomes Research Consortium.

[31] (2022). Real-world evaluation to facilitate adoption at scale. The AHSN Network.

